# Supplementary material for: Reconciling Mining with the Conservation of Cave Biodiversity: A Quantitative Baseline to Help Establish Conservation Priorities
Source: PLoS One. 2016 Dec 20;11(12):e0168348. doi: 10.1371/journal.pone.0168348 (PMC5173368; doi:10.1371/journal.pone.0168348)
Supplement: S1 Dataset — (ZIP) [file pone.0168348.s002.zip › Taxa/Serra Norte/SN_2013/N4E/Lista N4E-18.pdf]

CAVIDADE N4E-0018

Classe	Ordem	Fam/Outros	Gên/Outros	Espécie	S-U	U-S	Zon.
Annelida	Oligochaeta			jovem		1	E
Arachnida	Acari	(Oribatida)		jovem	X		
Arachnida	Araneae	Araneidae		jovem		X	E
Arachnida	Araneae	Caponiidae		jovem		X	E
Arachnida	Araneae	Corinnidae	*Creugas*	sp.1		1	E
Arachnida	Araneae	Ctenidae		jovem	1		
Arachnida	Araneae	Mysmenidae		jovem	X		
Arachnida	Araneae	Pholcidae	*Mesabolivar*	sp.1	X	X	E
Arachnida	Araneae	Salticidae		sp.	X		
Arachnida	Araneae	Scytodidae	*Scytodes*	sp.	X		
Arachnida	Araneae	Segestriidae	*Ariadna*	sp.	X		
Arachnida	Araneae	Theridiidae		sp.	X		
Arachnida	Araneae	Theridiosomatidae	*Ogulnius*	sp.1		X	E
Arachnida	Pseudoscorpiones	Chthoniidae		jovem		X	E
Chilopoda	Scolopendromorpha	Cryptopidae	*Cryptops*	jovem		1	E
Diplopoda	Polydesmida	Chelodesmidae		jovem		1	E
Diplopoda	Polydesmida	Fuhrmannodesmidae		jovem		X	E
Diplopoda	Polydesmida			sp.	X		
Diplopoda	Spirostreptida	Pseudonannolenidae	*Pseudonannolene*	jovem		1	E
Entognatha	Collembola	Entomobryidae		sp.4		X	E
Entognatha	Collembola	Entomobryidae		sp.6		X	E
Entognatha	Collembola	Paronellidae		sp.1		X	E
Entognatha	Collembola	Paronellidae		sp.4		X	E
Entognatha	Collembola			sp.	X		
Entognatha	Diplura	Campodeidae		sp.1		X	E
Entognatha	Diplura	Projapygidae		sp.1		X	E
Insecta	Blattodea			jovem		1	E
Insecta	Coleoptera	Scarabaeidae		sp.	X		
Insecta	Coleoptera	Staphylinidae		sp.57		X	E
Insecta	Diptera	Cecidomyiidae		sp.	X		
Insecta	Diptera	Culicidae		sp.	X		
Insecta	Diptera	Dolichopodidae		sp.	X		
Insecta	Diptera	Psychodidae	Psychodinae	sp.	X		
Insecta	Diptera	Psychodidae	*Sciopemyia*	*sordellii*	X	X	
Insecta	Diptera	Tipulidae	Tipulinae	sp.	X	X	E
Insecta	Heteroptera	Dipsocoroidea		sp.		X	E
Insecta	Heteroptera	Lygaeidae		sp.		X	E
Insecta	Heteroptera			sp.	X		
Insecta	Homoptera	Cixiidae		jovem		X	E
Insecta	Hymenoptera	Formicidae	*Camponotus*	atriceps	X		
Insecta	Hymenoptera	Formicidae	*Camponotus*	sp.1		X	E
Insecta	Hymenoptera	Formicidae	*Dolichoderus*	aff. *bispinosus*	X		
Insecta	Hymenoptera	Formicidae	*Gnamptogenys*	sp.1		X	E
Insecta	Hymenoptera	Formicidae	*Gnamptogenys*	*striatula*	X		
Insecta	Isoptera			sp.		X	E
Insecta	Lepidoptera	Noctuoidea		sp.5		1	E
Insecta	Orthoptera	Phalangopsidae	*Paraclodes*	sp.	X		
Insecta	Plecoptera			sp.	X		
Insecta	Psocoptera	Epipsocidae	*Epipsocus*	sp.2		X	E
Insecta	Psocoptera	Epipsocidae	*Epipsocus*	sp.4		X	E
Insecta	Psocoptera	Psocomorpha		jovem	X		
Insecta	Psocoptera	Trogiomorpha		sp.	X		
Gastropoda	Pulmonata	Systrophiidae	*Happia*	sp.	X		
Malacostraca	Isopoda	Philosciidae		sp.2	X	X	E
Turbellaria	Tricladida			sp.1		1	E

Amphibia	Anura			sp.		1	P
Mammalia	Chiroptera			sp.	1	3	E/P
